# The Structural Evolution of Al_86_Ni_9_La_5_ Glassy Ribbons during Milling at Room and Cryogenic Temperatures

**DOI:** 10.3390/ma11101956

**Published:** 2018-10-12

**Authors:** Zhicheng Yan, Yan Liu, Shaopeng Pan, Yihua Hu, Jing Pang, Weimin Wang

**Affiliations:** 1 Key Laboratory for Liquid-Solid Structural Evolution and Processing of Materials, Ministry of Education, Shandong University, Jinan 250061, China; Rengaryzc@outlook.com (Z.Y.); Xurixiangyu@163.com (Y.H.); 2 College of Materials Science and Engineering, Shandong Jianzhu University, Jinan 250101, China; 3 College of Materials Science and Engineering, Taiyuan University of Technology, Taiyuan 030024, China; shaopengpan@163.com; 4 Qingdao Yunlu Advanced Materials Technology Company Limited, Lancun Town, Qingdao 266232, China; pangjing@yunlu.com.cn

**Keywords:** metallic glass, milling, crystallization, structural rejuvenation, shear band

## Abstract

Melt-spun metallic Al_86_Ni_9_La_5_ glassy ribbons solidified at different circumferential speeds (*S*_c_) were subjected to high-energy ball milling at room and cryogenic temperatures. Crystallization induced by milling was found in the Al_86_Ni_9_La_5_ solidified at lower circumferential speed (*S*_c_ = 14.7 m/s), while the Al_86_Ni_9_La_5_ with *S*_c_ = 36.6 m/s kept amorphous. Besides, a trend of structural rejuvenation during milling process was observed, as the onset temperatures (*T*_x1_, *T*_x2_) and the crystallization enthalpies (Δ*H*_1_, Δ*H*_2_) first decreased and then increased along with the milling time. We explored the structural origin of crystallization by ab initio molecular dynamic simulations and found that the tricapped trigonal prism (TTP) Ni-centered clusters with a higher frequency in samples solidified at a lower cooling rate, which tend to link into medium-range orders (MROs), may promote crystallization by initiating the shear bands during milling. Based on the deformation mechanism and crush of metallic glasses, we presented a qualitative model to explain the structural rejuvenation during milling.

## 1. Introduction

Ball milling is a method for materials synthesis by solid-state reaction. In this process, elemental or alloy powders are subjected to highly energetic impact forces, resulting in the formation of new phases. Various kinds of defects and chemical disorder are introduced into crystalline solids during milling [1,2,3]. It was first performed on Fe-based amorphous alloys by Trudeau et al. to explore the structural evolution during milling [4]. They found that the crystallization can be induced by high-energy mechanical deformation, which is different from the on due to a thermal process. Poon et al. [5] found that the milling-induced crystallization is sensitive to the chemical composition in Al-based metallic glass. Milling condition also plays an important role on the structural evolution of metallic glass. Fan et al. [6,7] respectively milled Fe_80_B_20_ glassy ribbons with low and high milling energy, and found that the Fe_80_B_20_ glassy ribbons became more disordered after low-energy milling and more ordered after high-energy milling. At cryogenic temperature, the mechanical behavior of Ti-based metallic glass is found quite different with that at room temperature [8]. Similar phenomenon is found in Ni-based metallic glass [9]. So it is reasonable to believe that the structural evolution of metallic glass milled at cryogenic temperature will differ from that milled at room temperature. However, there are few reports about it.

At low temperatures (compared with the glass transition temperature *T*_g_) and high stress level, the deformation of the metallic glass is localized in shear bands [10], the formation of which has important practical consequences for the strength, ductility and toughness of metallic glasses. Chen et al. found that the deformation-introduced crystallization occurs within the shear bands [11]. During milling, shear bands are induced by severe deformation and play an important role in crystallization [12]. It was reported that the precursor structure influences a lot on the initiation and propagation of the shear bands [13], which is supposed to influence the crystallization behavior during milling.

Besides crystallization, structural rejuvenation can also be induced in metallic glass by severe plastic deformation [14]. And the structural evolution of metallic glasses generally shows a monotonous tendency (relaxation or rejuvenation) along with the milling time, the observation intervals of which is at least half an hour [4,6,7,15]. There comes a question that whether this monotonicity just an observation result under a long observation interval, since an exception was found that crystallization and rejuvenation appear alternately in Zr_66.7_Cu_33.3_ metallic glass during the milling process [16]. A shorter observation interval is needed for further exploration.

In this paper, we study the effects of the precursor structure and the milling temperature on the structural evolution of Al_86_Ni_9_La_5_ metallic glassy ribbons during 21-min-milling and the relationship between matrix structure, shear bands and crystallization induced by milling.

## 2. Materials and Methods

The ingot of Al_86_Ni_9_La_5_ was prepared by high frequency induction-melting pure Al, Ni, and La materials (purity > 99.9 wt.%) in the air. The ribbons were prepared by a single-roller melt-spinning equipment in the air at the circumferential speeds (Sc) of 14.7 and 36.6 m/s, the length, width and thickness of which are about 50 cm, 2 mm, 25 μm respectively. Then the ribbons were cut into pieces with the length of 10 mm and divided into two groups for milling at cryogenic temperature (77 K, CT) and room temperature (298 K, RT). Before being milled at cryogenic temperature, the ribbons were pre-frozen by liquid nitrogen for 10 min. No pretreatment was performed on the ribbons before being milled at room temperature. Both milling processes lasted 6, 12, 21 min. The ball-to-powder ratio is 20:1 and the milling frequency is 20 Hz. We tore as-cast ribbons for more details of the microstructure on their cross sections. In addition, to obtain the details of the microstructure on the surface of milled samples in SEM investigation, especially the details of shear bands, we polarized them in 3.5 wt.% NaCl solution with a sweep speed of 5 mV/s.

An X-ray diffractometer (XRD) (Rigaku, Tokyo, Japan) with a D/Max-rB diffractometer with Cu Kα radiation was used to characterize the structure. The cross section fracture morphology of as-spun Al_86_Ni_9_La_5_ ribbons after tearing and the surface morphology of the samples cryomilled for 12 min were examined by scanning electron microscopy (SEM, HITACHI SU-70) (HITACHI, Tokyo, Japan). The thermal analysis of the samples milled for different times was performed by using a differential scanning calorimetry (DSC) [17] (Netzsch DSC 404) (Netzsch, Selb, Germany) under an Ar atmosphere from room temperature to 773 K with a heating rate of 10 K/min.

AIMD simulations were analyzed on the basis of the density functional theory using the Vienna ab initio simulation package (VASP) [18] by implementing the projector augmented wave method [19]. All dynamical simulations were carried out in a canonical ensemble NVT through Nosé thermostat to control temperature [20]. Cubic cells containing 100 atoms with a periodic boundary condition were used to simulate the liquid and glassy Al_86_Ni_9_La_5_ alloys. The initial structures were equilibrated at 2000 K, well above the melting temperature of the alloy. After sufficiently relaxed for 4000 steps, the melt was cooled to 300 K at the rate of 10^15^ K/s and 10^13^ K/s to produce amorphous solids. At each temperature, the last 3000 configurations were used for analysis. The data of 300 K glasses were chosen for the structure analysis.

## 3. Results and Discussion

### 3.1. Crystallization and Crush in Milling

**Figure 1:**
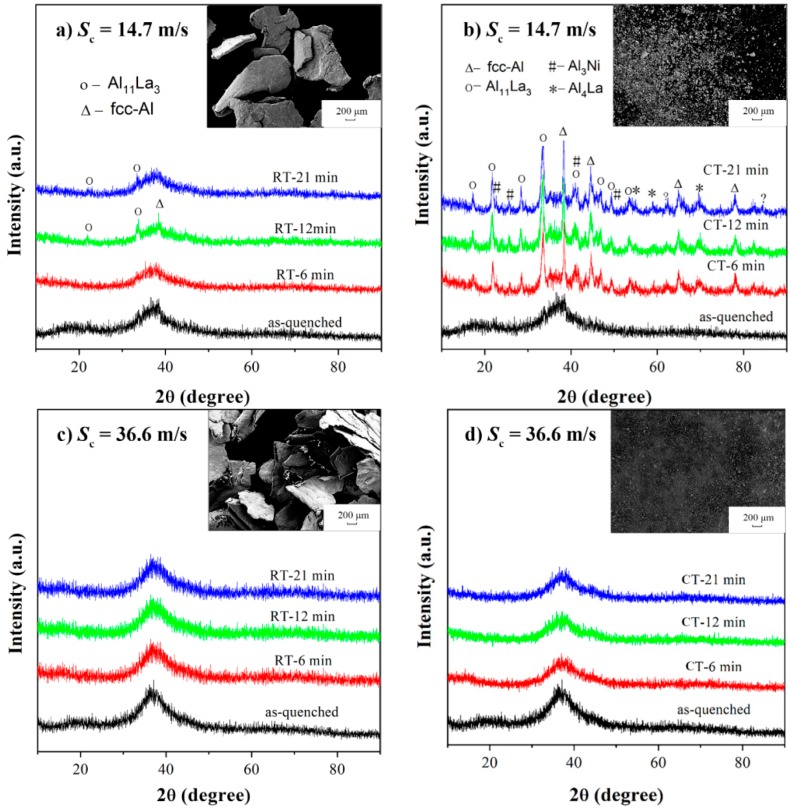
The XRD patterns of milled Al_86_Ni_9_La_5_ samples with different *S*_c_ and milling temperatures: (**a**) RT, *S*_c_ = 14.7 m/s; (**b**) CT, *S*_c_ = 14.7 m/s; (**c**) RT, *S*_c_ = 36.6 m/s; (**d**) CT, *S*_c_ = 36.6 m/s. The inset of each pattern is the SEM image of the corresponding samples after 21 min of milling.

Figure 1 shows the milling time dependent XRD patterns of the milled amorphous Al_86_Ni_9_La_5_ samples, which are respectively solidified at two circumferential speeds (*S*_c_ = 14.7 and 36.6 m/s) at room (298 K, RT) and cryogenic (77 K, CT) temperatures. For the samples with *S*_c_ = 14.7 m/s, the crystalline peaks corresponding to the fcc-Al and Al-La phase appear after 12 min of milling at room temperature, but only Al-La phase remains after 21 min (Figure 1a). After cryomilling, more crystalline peaks indexed as fcc-Al, Al-La and Al-Ni phases with higher intensities appear at the beginning (*t*_m_ = 6 min) and none of them disappears latter (Figure 1b). These XRD patterns indicate that crystallization occurs more significantly during cryomilling. For the samples with *S*_c_ = 36.6 m/s, no matter milled at room temperature (Figure 1c) or cryogenic temperature (Figure 1d), the XRD patterns show a fully amorphous characteristic with all measured milling times, indicating that the milling-induced crystallization is inhibited in the amorphous Al_86_Ni_9_La_5_ samples with a higher *S*_c_. A pre-peak locates at 20° beside the main amorphous peak in XRD patterns of as-spun samples, indicating the formation of medium-range orders (MROs) with solute-centered clusters according to Li’s work [21].

In addition, after 21-min-milling, the Al_86_Ni_9_La_5_ samples with *S*_c_ = 14.7 m/s are crushed into pieces with a larger size than those with *S*_c_ = 36.6 m/s, no matter at room or cryogenic temperature (insets of Figure 1), indicating a higher toughness of the former. Meanwhile, the size of the samples milled at room temperature (millimeter scale) are larger than that milled at cryogenic temperature (micrometer scale). At such low temperature, the shear transformation zone (STZ) activation is difficult and plastic deformation in MGs is negligible [22], which causes the observed brittleness.

**Figure 2:**
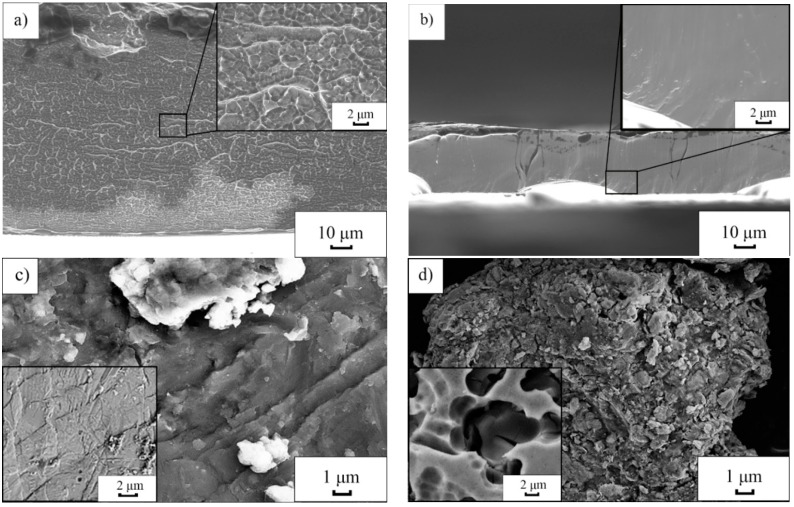
Micrographs of the fracture surface of as-spun Al_86_Ni_9_La_5_ ribbons with different *S*_c_: (**a**) *S*_c_ = 14.7 m/s; (**b**) *S*_c_ = 36.6 m/s. SEM images of the Al_86_Ni_9_La_5_ powders with different *S*_c_ after 12 min cryomilling: (**c**) *S*_c_ = 14.7 m/s; (**d**) *S*_c_ = 36.6 m/s. The insets in (**c**,**d**) are the SEM images of the 12-min-cryomilled powders after being polarized in NaCl solution with a sweep speed of 5 mV/s.

The cross section fracture morphology of as-spun Al_86_Ni_9_La_5_ ribbons after tearing and surface morphology of the particles after 12-min-cryomilling are shown in Figure 2. After tearing, there are more visible vein-like patterns on the fracture surface of as-spun ribbon with *S*_c_ = 14.7 m/s than that with *S*_c_ = 36.6 m/s under the same magnification (Figure 2a,b), indicating a higher toughness of the former according to the earlier fracture argument [23]. After 12-min-cryomilling, the “slip steps” associated with shear bands can be obviously observed on the surface of Al_86_Ni_9_La_5_ particles with *S*_c_ = 14.7 m/s (Figure 2c), while on the surface of particles with *S*_c_ = 36.6 m/s, there is no “slip step” but many micro-cracks (Figure 2d). The 12-min-cryomilled samples are polarized in NaCl solution with a sweep speed of 5 mV/s. After polarization, on the surface of the particles with *S*_c_ = 14.7 m/s, the region between the shear bands is rough (inset of Figure 2c), indicating a typical crystalline character; while the surface of the particles with *S*_c_ = 36.6 m/s is smooth and bears no shear band but pits and micro-cracks (inset of Figure 2d), showing a typical amorphous character.

Unlike the crystalline materials, metallic glasses do not exhibit long-range translational symmetry and consequently have no slip system but shear bands during deformation [24]. At low temperature (compared with the glass transition temperature *T*_g_) and a high stress level, the structural inhomogeneity and defects in amorphous alloys can introduce the stress concentrations and thus promote the shear band initiation, which helps absorb the energy during deformation and enhance the toughness [25]. It is understood that Al_86_Ni_9_La_5_ samples with *S*_c_ = 14.7 m/s, where the shear band operation is performed more easily than that of *S*_c_ = 36.6 m/s during milling (Figure 2), has a higher toughness. According to the work of Chen [11], the atomic rearrangement in shear bands will make the atoms shift towards more stable position and cause the crystallization. The fact that no crystallization is found in Al_86_Ni_9_La_5_ with *S*_c_ = 36.6 m/s during milling can be ascribed to the short of shear band, the underlying reason of which is related to the structure. Moreover, as the crystallization is an exothermic process in metallic glasses, the crystallization kinetic is greatly promoted in the Al_86_Ni_9_La_5_ glass with *S*_c_ = 14.7 m/s at a cryogenic temperature during milling, compared with that at room temperature (Figure 1a,b).

### 3.2. Atomic Structural Simulation

**Figure 3:**
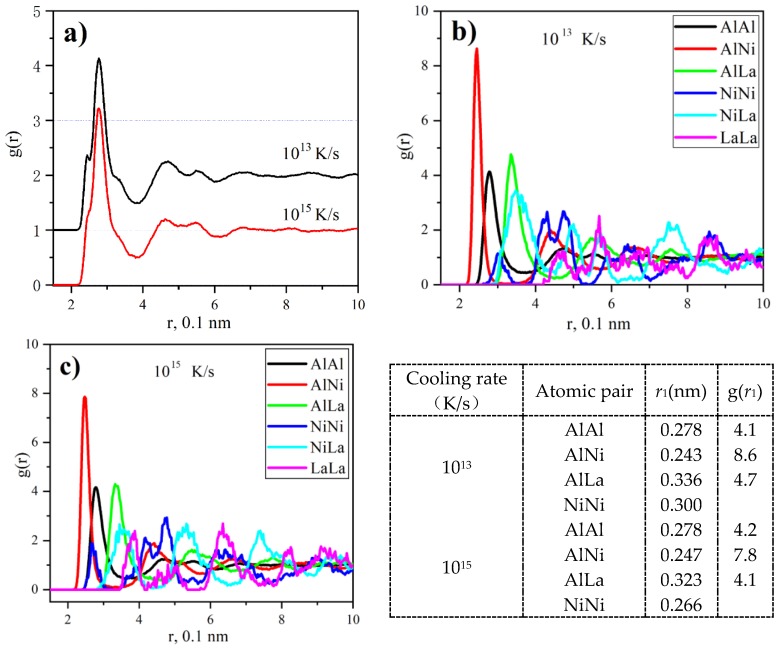
AIMD simulation of the Al_86_Ni_9_La_5_ alloy cooled down from 1500 K to 300 K at the rate of 10^15^ K/s and 10^13^ K/s: (**a**) total pair distribution function *g*(*r*); (**b**) partial *g*(*r*) with the cooling rate of 10^15^ K/s; (**c**) partial *g*(*r*) with the cooling rate of 10^13^ K/s, the inset contains the parameters of the atomic pairs in (**b**,**c**).

To explore the structure origin of the crystallization and crush behavior of samples with different *S*_c_, we tracked the influence of cooling rate on the structure of Al_86_Ni_9_La_5_ glass via ab initio molecular dynamic simulations, the results of which are shown in Figure 3.

Figure 3a gives the total pair distribution function *g*(*r*) of Al_86_Ni_9_La_5_ glass with cooling rates of 10^13^ and 10^15^ K/s. The splitting of the second peak can be found in both curves, showing a typical glassy character [26,27]. A shoulder peak exists at the left side of the main peak, which is similar to the simulated Al-Zr glass [28]. Figure 3b,c give the partial pair distribution functions *g*(*r*) of Al_86_Ni_9_La_5_ metallic glasses solidified at cooling rates of 10^13^ K/s and 10^15^ K/s, respectively. The first peak position *r*_1_ (i.e., the bond length) and corresponding *g*(*r*_1_) (i.e., the amount of band) of different atom pairs are listed in the inset of Figure 3. Because of the strong orbital hybridization of the 3d-states of Ni with the 3 s and 3 *p*-states of Al [29], the Al-Ni bond appears in a covalent bond rather than a metallic bond and bears a relatively short bond length. Thus, it is expected the existence of the shoulder peak (Figure 3a). According to the work of Sheng [30], the Al-based amorphous alloys are characterized by solute-centered quasi-equivalent clusters, where the solute atom is surrounded by solvent atoms in the nearest-neighbor shell. These solute-centered clusters are connected into medium-range orders (MROs) by sharing the Al atoms. Therefore, the shorter bond length and higher *g*_Al-Ni_(*r*_1_) of Al-Ni bonds reflects more MROs in the Al_86_Ni_9_La_5_ glass with the cooling rates of 10^15^ K/s (inset of Figure 3).

**Figure 4:**
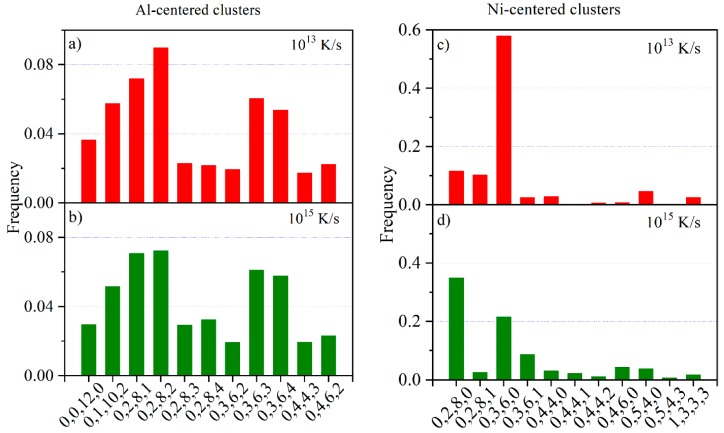
Distribution of Al- and Ni-centered clusters in Al_86_Ni_9_La_5_ glasses with two cooling rates of 10^15^ K/s and 10^13^ K/s: (**a**) 10^13^ K/s, Al-centered clusters; (**b**) 10^15^ K/s, Al-centered clusters; (**c**) 10^13^ K/s, Ni-centered clusters; (**d**) 10^15^ K/s, Ni-centered clusters.

The frequency of Al-centered and Ni-centered clusters in Al_86_Ni_9_La_5_ glasses with different cooling rates is given in Figure 4. The Voronoi polyhedron analysis is used to characterize the clusters. The Voronoi polyhedron index is shown as <*n*_3_ *n*_4_ *n*_5_ *n*_6_>, where *n*_i_ denotes the number of *i*-edged faces of the Voronoi polyhedron. Among the Al-centered clusters, the <0 0 12 0> icosahedral clusters and the distorted ones like <0 1 10 2>, <0 2 8 1> and <0 2 8 2> occupy a large proportion and keep almost unchanged with changing the cooling rate (Figure 4a,b). Among the Ni-centered clusters, the <0 3 6 0> regular tricapped trigonal prism (TTP) clusters, which tend to link into MROs as a framework in the glass matrix [31], possess the highest frequency when the cooling rate is 10^13^ K/s (Figure 4c). While under a higher cooling rate (10^15^ K/s), the dominated unit is <0 2 8 0> distorted TTP cluster rather than <0 3 6 0> regular TTP cluster (Figure 4d). The <0 2 8 0> cluster, which is evolved from <0 3 6 0> TTP by capturing one atom, can hardly link into MROs [32]. According to the work of Hufnagel [13], the MROs can help initialize the shear bands and improve toughness of a metallic glass. Therefore, in the Al_86_Ni_9_La_5_ metallic glass with a lower cooling rate, the higher frequency of <0 3 6 0> Ni-centered clusters and consequently more MROs are expected to be the structure origin of the more easily performed shear band operation during milling (Figure 2).

### 3.3. DSC Analysis and Structural Rejuvenation in Milling

**Figure 5:**
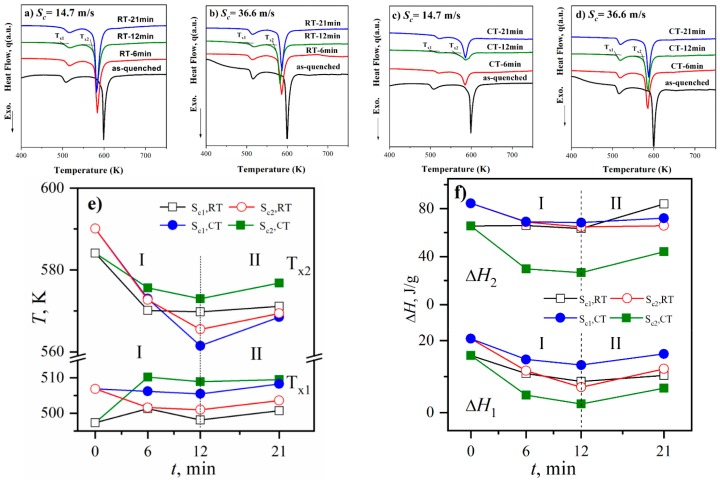
DSC curves of the milled Al_86_Ni_9_La_5_ samples with different *S*_c_ and different milling temperatures: (**a**) RT, *S*_c_ = 14.7 m/s; (**b**) RT, *S*_c_ = 36.6 m/s; (**c**) CT, *S*_c_ = 14.7 m/s; (**d**) CT, *S*_c_ = 36.6 m/s. The onset temperature (*T*_x1_, *T*_x2_) and crystallization enthalpy (Δ*H*_1_, Δ*H*_2_) calculated from the DSC curves are performed as a function of milling time in (**e**,**f**), where *S*_c1_ represents *S*_c_ = 14.7 m/s and *S*_c2_ represents *S*_c_ = 36.6 m/s.

Figure 5 presents the DSC curves of the milled Al_86_Ni_9_La_5_ samples as a function of milling time together with the correlative thermodynamic parameters are performed. In each DSC curve, there are two exothermic peaks locating at about 500 and 600 K (Figure 5a–d). Generally, the first exothermic peak on the DSC curves of Al-TM-RE metallic glasses is associated with the formation of fcc-Al nanocrystals and the second one is associated with the formation of intermetallic compound phases [33]. The onset temperatures (*T*_x1_, *T*_x2_) and the crystallization enthalpies (Δ*H*_1_, Δ*H*_2_) deduced from the two exothermic peaks can characterize the structural evolution during milling (Figure 5e,f). For a metallic glass, a lower value of *T*_x_ means a better crystallization kinetic. Meantime, a higher value of Δ*H* means a higher disordering degree of the glass.

As the milling time *t*_m_ goes from 0 to 21 min, the *T*_x2_, Δ*H*_1_ and Δ*H*_2_ of the samples with *S*_c_ = 14.7 and 36.6 m/s tend to decline first and then rise, the process of which can be divided into two stages (I and II) by the turning point at *t*_m_ = 12 min. In Stage I, the decline of *T*_x2_ with increasing *t*_m_ shows that it becomes easier to form Al-La and Al-Ni phases, and the decline of Δ*H*_1_ and Δ*H*_2_ indicates the relaxation of the amorphous structure during milling. Moreover, the decrease amount of *T*_x2_, Δ*H*_1_ and Δ*H*_2_ in the first 6 min is significantly larger than in the second 6 min, the changing trend of which is same with the size reduction of samples during milling [34]. In Stage II, the *T*_x2_, Δ*H*_1_ and Δ*H*_2_ increase with increasing *t*_m_, indicating that the structure is turning back to be disordered and structural rejuvenation occurs during milling. The *T*_x1_ of the samples with *S*_c_ = 36.6 m/s has the same changing trend with the *T*_x2_, while the *T*_x1_ of samples with *S*_c_ = 14.7 m/s shows an opposite tendency at the beginning 6 min. The mechanism for the abnormal increment of *T*_x1_ with *S*_c_ = 14.7 m/s is unclear but believed to be relevant with the pre-existing fcc-Al nuclei in Al-based glass with a low *S*_c_ [35], the role of which is complex during milling.

**Figure 6:**
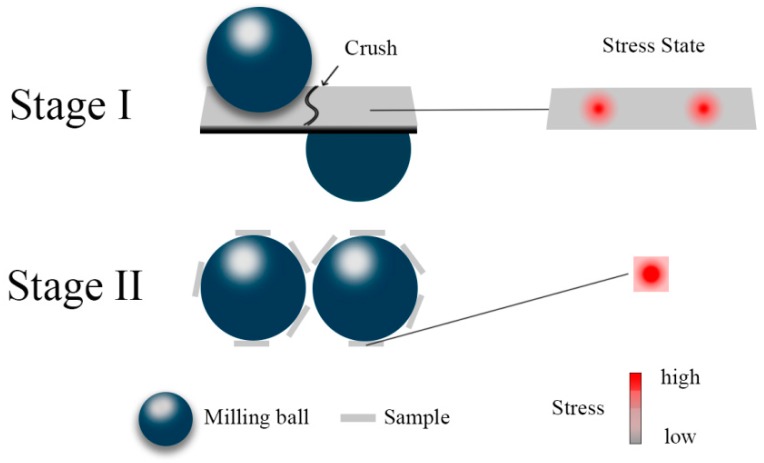
Schematic drawing of the different stress states of samples in Stage I and Stage II during milling.

The schematic drawing of Stage I and II is shown in Figure 6. In amorphous alloys, there are regions with a high stress and a low symmetry, which are defined as structural defects [36]. In Stage I, the Al_86_Ni_9_La_5_ ribbons are crushed into pieces because of the heterogeneous stress in a relatively big area. The structural defects inside are therefore exposed to the surface and relaxed. Besides, the atomic rearrangements within the milling-induced shear bands would cause the crystallization [11]. These factors contribute to the decline of the *T*_x_ and Δ*H* of milled samples (Figure 5). In Stage II, the size reduction of samples gradually slows down and the samples undergo more plastic deformation rather than crush. Especially at cryogenic temperature (77 K), the stress on tiny particles can be approximately considered homogeneous because the contact area between the tiny particles and milling balls nearly takes up the whole projected area of the former. According to Heggen’s work [37], the structure of a metallic glass tends to be disordered under strong deformation. In short words, the crush and plastic deformation coexist during milling process: at the beginning, the effect of the crush is dominated and results in structural relaxation and possible crystallization; under further milling, the effect of the plastic deformation gradually takes the lead and the structural rejuvenation occurs.

## 4. Conclusions

The structural evolution of Al_86_Ni_9_La_5_ metallic glassy ribbons in milling process was studied by experimental and computational techniques. We find:

1. Crystallization occurs in the Al_86_Ni_9_La_5_ metallic glass with *S*_c_ = 14.7 m/s during milling at cryogenic temperature, which is weakened at room temperature. As a contrast, no crystallization has been checked in the milled or cryomilled samples with *S*_c_ = 33.6 m/s. The Al_86_Ni_9_La_5_ metallic glass with *S*_c_ = 14.7 m/s bears more shear bands than that with *S*_c_ = 36.6 m/s during milling and the crystallization in former is closely related with the shear bands.
2. Besides icosahedral Al-centered clusters, the regular and distorted tricapped trigonal prism (TTP) Ni-centered clusters are dominant in Al_86_Ni_9_La_5_ glass. With decreasing the cooling rate, the frequency of Al-centered cluster keeps almost unchanged, while the frequency of Ni-centered TTP cluster increases drastically. The higher frequency of regular Ni-centered TTP clusters makes them easier to link into MROs and affects the initiation and propagation of shear bands as well as the possible crystallization.
3. The changing trend of the onset temperatures (*T*_x1_, *T*_x2_) and crystallization enthalpies (Δ*H*_1_, Δ*H*_2_) reflects the existence of structural rejuvenation during milling. At the beginning of the milling, the samples are crushed into pieces and the defects in the glass are consequently relaxed; after further milling, the size of samples is small enough compared with the milling ball and the stress state of the samples shifts from heterogeneous to homogeneous, which makes the structure more disordered and results in the structural rejuvenation.
